# An integrative mathematical model for timing treatment toxicity and Zeitgeber impact in colorectal cancer cells

**DOI:** 10.1038/s41540-023-00287-4

**Published:** 2023-06-23

**Authors:** Janina Hesse, Tim Müller, Angela Relógio

**Affiliations:** 1 Institute for Systems Medicine, Faculty of Human Medicine, MSH Medical School Hamburg, Hamburg, 20457 Germany; 2 Institute for Theoretical Biology (ITB), Charité – Universitätsmedizin Berlin, corporate member of Freie Universität Berlin, Humboldt-Universität zu Berlin, and Berlin Institute of Health, Berlin, 10117 Germany; 3 Molecular Cancer Research Center (MKFZ), Medical Department of Hematology, Oncology, and Tumor Immunology, Charité – Universitätsmedizin Berlin, corporate member of Freie Universität Berlin, Humboldt-Universität zu Berlin, and Berlin Institute of Health, Berlin, 10117 Germany

**Keywords:** Cancer, Computational biology and bioinformatics, Computer modelling

## Abstract

Increasing evidence points to a role of the circadian clock in the regulation of cancer hallmarks with a strong impact on the understanding and treatment of this disease. Anti-cancer treatment can be personalized considering treatment timing. Here we present a new mathematical model based on data from three colorectal cancer cell lines and core-clock knock-outs, which couples the circadian and drug metabolism network, and that allows to determine toxicity profiles for a given drug and cell type. Moreover, this model integrates external Zeitgebers and thus may be used to fine-tune toxicity by using external factors, such as light, and therefore, to a certain extent, help fitting the endogenous rhythms of the patients to a defined clinic routine facilitating the implementation of time-dependent treatment in clinical practice.

## Introduction

A wide range of diseases are reportedly associated with the disruption of circadian rhythms, such as obesity, sleeping disorders, neurodegenerative diseases and cancer development^1,2^. To outgrowth in the body, cancer cells need to escape a series of safe keeping mechanisms known as the hallmarks of cancer^3^. Accumulating evidence points to a role for the circadian clock in regulating most, if not all, of these hallmarks^4^. Understanding the mechanisms that relate the circadian clock to cancer will help to develop new therapies or optimize existing ones.

Cancer is a major health problem, it caused death in about one out of six persons, and was the second most common cause of death worldwide in 2020. This number is expected to increase with the continuous aging of the population^5^. Colorectal cancer (CRC) is the third most common cancer worldwide. Estimates from 2020 point to 1.9 million incidence cases and 0.9 million deaths making CRC the second most deadly cancer type worldwide^6^. CRC occurs in the large intestine (colon) and the rectum of the gastrointestinal tract. Depending on the exact location, it is also referred to as large bowel cancer, colon cancer or rectal cancer. CRC affects mostly older patients, with a median age at diagnosis of 72 years in males and 75 years in females^7^. Many patients are treated with chemotherapy, during which they receive a drug that interferes with the mechanism of cell division, and thus induces cell death preferentially in fast-dividing cells. Fast dividing cells encompass not only cancer cells, but also, for example, cells in the gastrointestinal tract, which leads to side effects such as strong diarrhoea, particularly devastating for patients of old age. Chronotherapy aims to alleviate the side effects of anti-cancer treatment by aligning the drug exposure to the circadian time of the patient^8^, and several clinical studies reported an overall treatment improvement in cancer when considering drug administration time^9^. The desired timing where drug effectiveness is maximised and side effects minimized results from the regulation via the circadian clock over the expression of numerous genes including those responsible for drug metabolism^10^. In a clinical trial, in which patients received the drug irinotecan at different times of the day, an ideal time of treatment, for which patients showed the least severe side effects, could be identified for sex-specific patient groups^10^, this is also the case for other anti-cancer drugs^11^. For personalized treatment with irinotecan, we established previously a mathematical model, which relates circadian gene expression with the circadian profile of irinotecan toxicity^12^. The model combined a transcription-translation network of the core clock and of clock-regulated genes relevant for irinotecan with a model of irinotecan pharmacokinetics and -dynamics (PK-PD). Our previous work on SW480 and SW620 CRC cells showed different toxicity profiles, in particular a flatter circadian toxicity profile for the SW620 cell line with reduced circadian oscillations^12^.

The need to have a mathematical model that can potentially represent also other cells, and to predict cytotoxicity based on new datasets, as well as to allow for a fitting of cytotoxicity predictions based on the application of external Zeitgebers, motivated us to develop the chronotherapy model here presented, which is based on a transcription-translation network for the CRC cell lines, HCT116, as well as SW480 and SW620 cell lines. Compared to our previous mathematical model by Hesse et al. 2021^12^, we could improve the fit of the data by network refinements including new network connections. The fit quality improved the coefficient of determination from a R^2^ value of 0.23 (Hesse et al. 2021^12^) to a R^2^ value of 0.29 with the here proposed refinements (Supplementary Fig. 1). We also refined the PK-PD part of the model by including biologically motivated temporal effects of treatment on gene expression, such as an increase in UGT1A1 and a transient increase in apoptosis rate.

The refined model is able to fit, in addition to the HCT116 wild type (WT), also three different core-clock knock-outs (KOs), *PER2*^KO^, *NR1D1*^KO^, and *ARNTL*^KO^, with altered circadian oscillations.

In patients, an analogue altered circadian rhythm amplitude, and the expected reduced circadian oscillation of the toxicity, would make chronotherapy less effective. To alleviate this problem, the usage of external Zeitgebers (e.g. light, which can be used as phototherapy) is an interesting option, which may enhance circadian amplitudes of the core clock and thus impact toxicity profiles. In addition, external Zeitgebers may enable to manipulate the patients’ cytotoxicity curves, which would allow, for example, to fit a patient internal rhythm to a certain clinic schedule. We thus incorporated in our model the influence of external Zeitgebers, such as light, to the predicted toxicity profile. Our results show that our model, which can be fitted to different human CRC cell lines, also allows us to generate personalized cytotoxicity curves, which can be further fine-tuned via external Zeitgebers. Such an integrative approach using the individual biological data (here cell lines) allows for a personalization of treatment schedules, which may strongly benefit treatment outcome and that can be further adjusted, if need be, to implement an enhancement of the patients’ endogenous rhythms, as well as an integration in a realistic clinic treatment schedule, by using external cues to fine-tune the clock.

## Results

### A refined core-clock model for drug toxicity in colorectal cancer cells

**Fig. 1:**
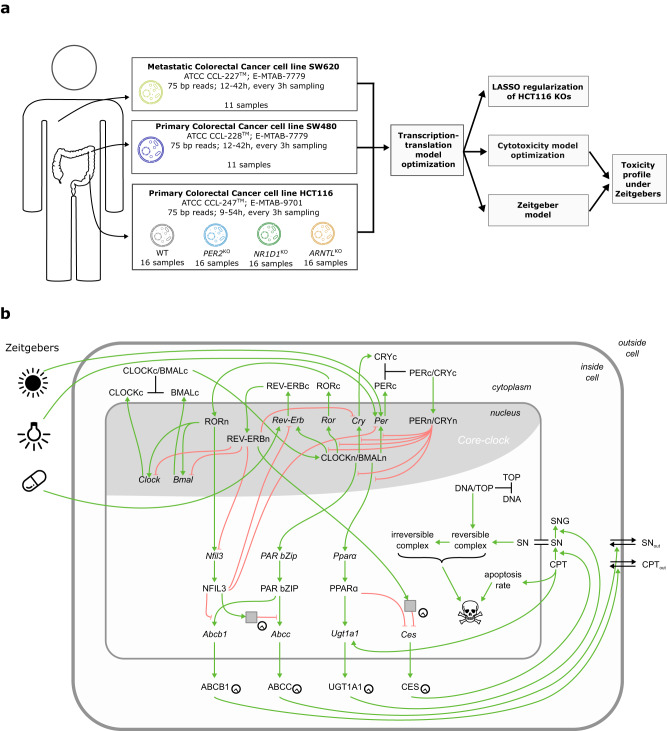
Based on the gene expression of different colorectal cancer cell lines fitted to an extended core-clock network, our model predicts toxicity profiles for irinotecan treatment. **a** Schematic representation of the work flow to generate the mathematical model. **b** Network of circadian regulation of toxicity. The extended core-clock model is connected to the PK-PD model by the expression of the ABC-transporters, *UGT1A1* and *CES2*. Interactions among the elements of the network are inhibitory (red arrows with flat arrowhead) or activating (green arrows), and complex formation is indicated by black lines. Grey boxes represent post-translational steps, elements marked with a clock implement circadian protein degradation. Irinotecan (CPT-11) and SN-38 transport through the outer cell membrane is denoted by double black lines with arrowheads; the concentration of SN-38 is assumed equal between nucleus and cytoplasm (double black line).

Scheduling anti-cancer drug administration over 24 h may critically impact treatment success in a patient-specific manner. We address personalization of anti-cancer treatment toxicity time for irinotecan, an anti-cancer drug widely used against digestive malignancies. For this we developed a mathematical model that links cellular pharmacokinetics and -dynamics (PK-PD) of irinotecan to a representation of the core clock, which together predict treatment toxicity based on circadian gene expression profiles, and further allow for a fine-tuning of cytotoxicity profiles using external Zeitgebers (Fig. 1).

We are particularly interested in heterogeneous circadian gene expression profiles to simulate the variety of expected circadian profiles within a patient cohort. The heterogeneity of different CRC cell lines is due to their pathological state rather large; even cell lines such as SW480 and SW620, which are derived from the same patient, but different tumour sites (primary tumour *vs*. a metastasis site, respectively), show strong differences in gene expression. We found an appropriate level of heterogeneity in a set of three core-clock KOs and the WT of the same CRC cell line (HCT116), as described below.

**Fig. 2:**
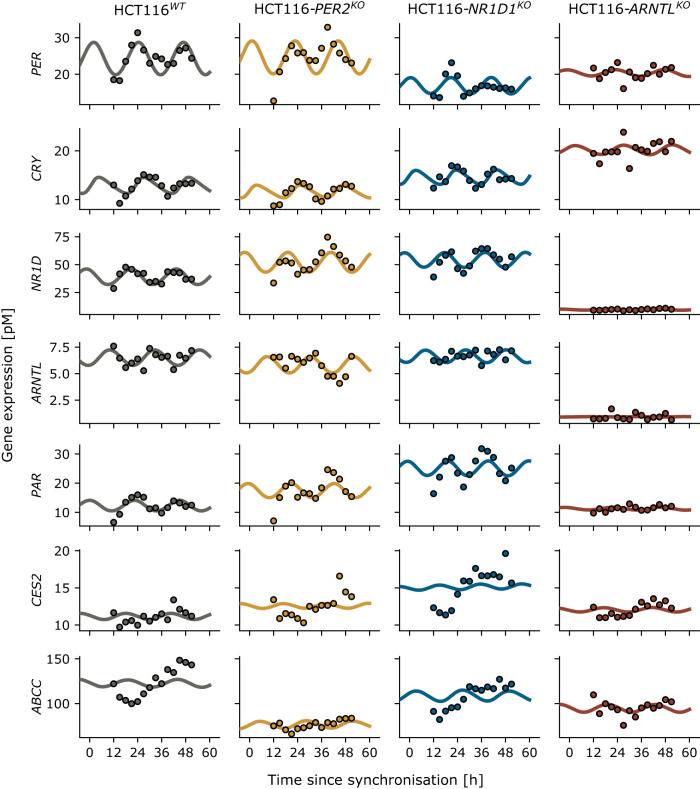
Changes to the core clock by knock-outs are less visible on pharmacologically relevant genes such as *CES2* and *ABCC*. Experimental time series (dots) and corresponding model fits (lines) for HCT116 wild type (WT) and knock-outs (KOs) of *PER2*, *NR1D1* and *ARNTL*. *PER*, *NR1D* and *CRY* denote the sum of the corresponding paralogous genes. Based on a model fit to the wild type (left column), the KO cell lines are fitted with minimal parameter divergence using a LASSO regularization.

The model used in this manuscript is based on a previous model by Hesse et al. 2021^12^. This new model combines a transcription-translation network for circadian gene expression with the pharmacokinetics and -dynamics of irinotecan, extending the previous model to other CRC cell lines including core-clock KOs. The model is improved to fit not only the previously used microarray data of the human CRC cell line SW480 and its metastatic counterpart, SW620^12^, but also for longer timeseries of RNA-seq data for the same cells (Supplementary Figs. 1 and 2), and for the human CRC cell line HCT116 and associated KO cell lines of the core-clock genes *PER2*, *NR1D1*, and *ARNTL* (Fig. 2).

The transcription-translation network and the PK-PD model part are linked by the expression of proteins related to irinotecan metabolism, including the translation of the four irinotecan-relevant mRNAs fitted by the network, *UGT1A1*, *CES2*, *ABCB* and *ABCC1*, which modulate cell death in the PK-PD associated network (Fig. 1b). As *TOP1* shows no significant oscillations at the gene expression level in CRC HCT116 cell lines, and the corresponding protein TOP1 is reported as constant in the Caco-2 human colorectal cancer cell line^13,14^, we removed *TOP1* from the transcription-translation network, and assumed a constant TOP1 protein expression for the PK-PD model part.

For the transcription-translation network, the model presented here refines the connections between network elements as compared to the previous model from Hesse et al. 2021^12^. In particular, *CES2* transcription is inhibited by PPARα^15^, and is only indirectly activated by NFIL3 via NR1D^16^, NFIL3 inhibits *PER*^17^, and NFIL3 and PAR compete for the same binding sites of the ABC-transporters^18^. We now consider post-translational steps with circadian protein degradation^19^, as an alternative to the post-transcriptional steps for *CES2* and *ABCC* (two for *ABCC* and three for *CES2* unidirectional activation steps) required in Hesse et al. 2021^12^ to fit the mRNA expression of *CES2* and *ABCC* (Fig. 1b). For the pharmacokinetics and -dynamics part, the current model replaces the treatment-induced phase-resetting of apoptosis modulation, as used in Hesse et al. 2021^12^, by a biologically supported increase in UGT1A1 with treatment time, and a transient increase in apoptosis modulation. The former is motivated by the observation of increased *UGT1A1* over time following treatment^20^, the latter by published work, which reports treatment-dependent alterations in genes that influence apoptosis, i.e., *DDIT4* (DNA-damage-inducible transcript 4 gene, also known as protein regulated in development and DNA damage response 1 (*REDD1*) gene), a negative regulator of mTOR that influences autophagy, shows a pronounced peak following irinotecan treatment start^21^. The full mathematical description of the model including the model equations with modifications marked in blue font colour is provided in the Supplementary Methods.

For the SW480 cell line, we compared fits to the RNA-seq data (sampled over 30 h) with the rescaled microarray data (sampled over 24 h) used in Hesse et al.^12^, see Supplementary Fig. 2.

For most genes, the Counts Per Million (CPM) values for the CRC cell lines HCT116 WT, *PER2*^KO^, *NR1D1*^KO^, and *ARNTL*^KO^, as well as SW480 lied within the same order of magnitude (maximal difference three-fold). The noteworthy exception is *UGT1A1*, responsible for drug removal, which was in the HCT116 cell lines very low expressed, in agreement with the literature^22^. The RNA-seq datasets of HCT116 WT, *PER2*^KO^, *NR1D1*^KO^, and *ARNTL*^KO^, as well as SW480 showed consistent drifts, or linear trends, in addition to the actual oscillation, that were not observed in the SW480 microarray data (for SW480 see Supplementary Fig. 2, drifts were particularly pronounced for the mRNAs of *RORc*, *PPARα* and *CES2*). Genes with a linear trend in the RNA-seq data were still fitted in reasonable agreement with the microarray data. This highlights the potential of the model to uncover hidden oscillations in the data. As the microarray data was measured at an earlier time point and for a shorter time interval as compared to the SW480 RNA-seq data, these linear trends may hint at an underlying adaptation to the fresh cell culture media used for synchronization only visible in the longer time series of the RNA-seq data.

### Paralog compensation contribute to the robustness of the circadian clock in CRC cells

**Fig. 3:**
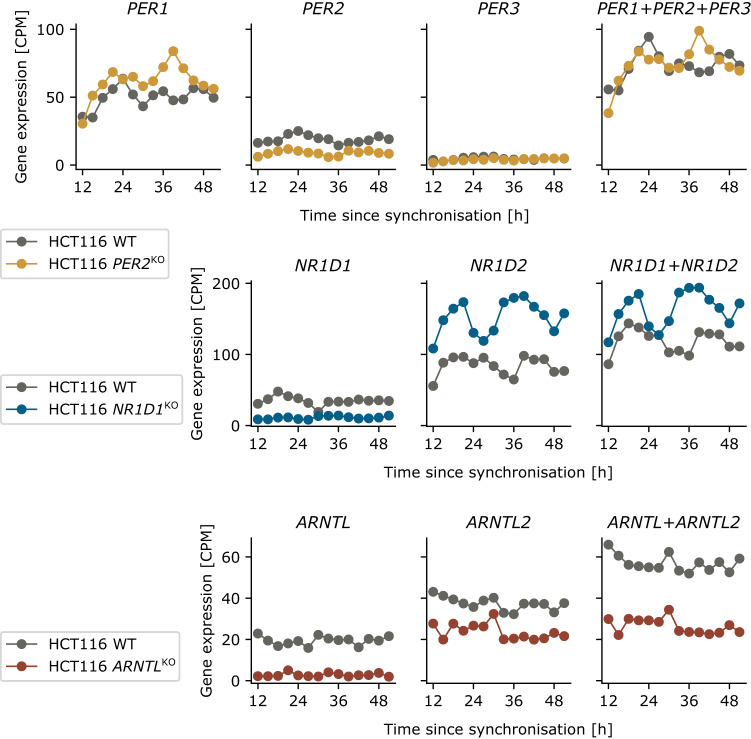
Paralog compensation in HCT116^KO^ cell lines by upregulation of paralogous genes. The HCT116 *PER2*^KO^ cell line shows an increase in *PER1* which seems to counterbalance the reduction in *PER2*, such that the sum of *PER1*, *PER2* and *PER3* gene expression remains constant. Analogue observations can be made for *NR1D1* and *NR1D2* in the *NR1D1*^KO^, but not *ARNTL* and *ARNTL2* in the *ARNTL*^KO^.

Our data shows paralog compensation for HCT116 cells, i.e. downregulation of one paralog leads to the upregulation of another, thereby maintaining the overall sum of expression profiles almost unchanged, in agreement with previous reports on other cell types^23^. In the *PER2*^KO^ cells, *PER1* is upregulated, likewise in the *NR1D1*^KO^ cells *NR1D2* is upregulated (Fig. 3). In contrast, *ARNTL1* and *ARNTL2* do not show compensation, *ARNTL1* KO leads to a downregulation also in *ARNTL2* in HCT116 *ARNTL1*^KO^ cells (Fig. 3). As we observed paralog compensation for *PER2* and *NR1D1*, we lumped certain paralogs within the core clock into one variable of the dynamical model, i.e. the dynamical variable *PER* is fitted to the sum of the expression data of *PER1*, *PER2* and *PER3*. Likewise, *NR1D* models the sum of *NR1D1* and *NR1D2*, and *CRY* models the sum of *CRY1* and *CRY2*. Such comparisons are possible because the used RNA-seq data is quantitative. We observed more stable characteristics with regard to gene expression level and oscillation amplitude in *PER* and *CRY* as compared to *NR1D* (with only partial paralog compensation, see Fig. 3) and *ARNTL* (without paralog compensation), see Fig. 2. This supports previously published data showing that paralog compensation enhances the robustness of the core clock^23^.

### The effect of core-clock KOs in HCT116 cells can be simulated by altering a subset of network parameters

As KO cell lines were derived with defined changes from the WT (single gene KOs), they should resemble the wild-type cell line, with strong differences in only few aspects of the gene regulatory network, which try to compensate for the gene loss. Regularization of the model fit resulted in a small subset of strongly deviating parameters that may give a hint as to which biological processes were altered in the KO as compared to the WT cell lines.

Our transcription-translation network models the interaction between genes, and could be fitted to all CRC cell lines here tested, including the three HCT116 core-clock KO cell lines (Fig. 2, Supplementary Fig. 2). The large number of model parameters and the restricted amount of data made the fit under-determined; a fit of KO cell lines that optimizes a cost function on the squared error between data and model simulation, i.e. that minimizes the distance between data and simulation, differs from the fitted WT scenario in several model parameters. Arguably, gene expression differs in the KO cell lines compared to the WT for two related reasons, the technical KO itself, and the biological counter strategies: the KO of a given gene changes the gene expression of all its direct or indirect target genes. The biological system aims to counter these altered expressions by selected changes to the gene network, thus partly recovering functional gene expression as observed in the WT condition. We assumed that these changes to the network occur in only a few parameters, but that the associated parameter values can vary strongly. Technically, this was implemented as a fit of the KO cell lines with LASSO regularization, which minimizes the parameter divergence between KO and WT. A penalty on the divergence of parameters from the WT parameters, i.e. an additive cost-related to the absolute difference of the new parameter value and the WT parameter value (see Methods), ensured that only few parameters of the new fit differ strongly from the WT scenario. LASSO regularization is appropriate for parameter selection, i.e. to identify which parameters differ most strongly between WT and KO.

**Fig. 4:**
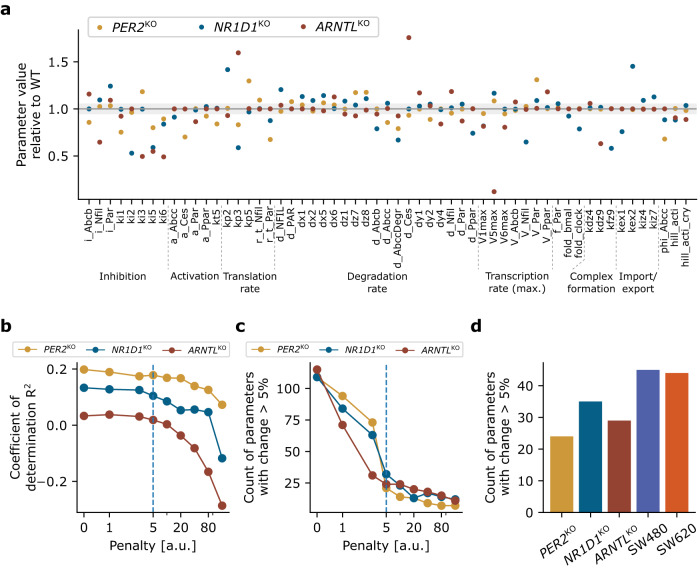
Compared to the HCT116 WT, the transcription-translation networks of the KOs show reduced interactions in the core clock, and are more similar to the WT than other CRC cell lines (SW480 and SW620). **a** Plotted are the parameters with values diverging from the WT values by more than 5% in at least one KO cell line. Grey region corresponds to maximal 5% change. **b**, **c** Choice of penalty for regularization. The penalty of *λ* = 5 (vertical dashed line) was chosen such that the parameter deviation of the KO cell lines from the WT was substantially reduced compared to the case without regularization, i.e. a penalty of zero (**b**), while the model fit quality (measured via the coefficient of determination *R*^2^) was not too strongly reduced (**c**). **d** Difference to WT is smaller for the three KO cell lines than for the SW480 and SW620 cell lines.

For the interpretation of the results, we considered parameters that differ 5% or less from the WT as not being relevantly changed (Fig. 4a). The number of parameters that vary more than 5% decreased with the penalty term *λ*, thus making the KO models more similar to the WT (Fig. 4b). However, larger penalty terms also reduced the quality of the fit (Fig. 4c). We aimed for a balance between both observations, thus the penalty term was chosen as *λ* = 5 such that the number of altered parameters was significantly reduced without reducing the model fit quality below 50% of the coefficient of determination R^2^ without regularization, see Fig. 4b, c. Penalty values larger than 5 reduced the number of parameters that vary more than 5% by not more than 20 compared to a count of over 100 parameters in the fit without regularization (Fig. 4b).

Our data showed that about half of the parameters is altered by more than 5% in at least one KO, see Fig. 4a. Overall, we observed a weakening of the interactions within the core clock. On the one hand, weaker interactions result from a reduction of the interaction strength, visible in Fig. 4a as those activation and inhibition rates with values below 1. In particular, all KOs showed a change in the inhibition of *ARNTL* and *CLOCK* (model parameters *k*_*i5*_ and *k*_*i6*_ are below 1 for all KOs, i.e. reduced compared to WT). On the other hand, weaker interactions result from an upregulation of nuclear protein degradation (model parameters *d*_*x1*_, *d*_*x2*_, *d*_*x5*_ and *d*_*x6*_ tend to be larger than 1, i.e. increased compared to WT). Thereby, faster degradation implies faster removal and thus a reduced amount of nuclear proteins in the KO cell lines compared to the wild type, and as the amount of nuclear proteins regulate the translation of other genes, the reduced amount of nuclear proteins can be interpreted as a reduced interaction between core-clock genes. These changes seem to hint at an overall reduced interaction between the core-clock elements of the KO cell lines compared to the WT. These results are in line with previous observations that under certain conditions, such as a perturbation of the clock due to external or internal factors, the interaction strength of the core-clock network is affected^24,25^.

The regularized fits to the KO cell lines fit the KO data nearly as good as non-regularized fits (Fig. 4b, compare R^2^ values at penalty 0, i.e. no regularization, with R^2^ values at penalty 5), and the fits do not resemble the WT data (negative R^2^ values result from a comparison of the KO model fits to WT data). To evaluate whether the transcription-translation network of the HCT116 KO cell lines is more similar to their WT as compared to other cell lines, we fit two different CRC cell lines, SW480 and SW620, using the same regularization of the parameters based on the HCT116 WT as for the KOs. In agreement with our assumption that the HCT116 KO cell lines show a larger similarity to the HCT116 WT as compared to other cell lines, we find stronger parameter deviations for the SW480 and SW620 cell lines compared to the HCT116 KO cell lines (Fig. 4d). This suggests that indeed the network of the HCT116 WT compared to that of the SW480 and SW620 cell lines is more different than compared to the HCT116 KOs, thus supporting our assumption that the KO cell lines show networks similar to the WT.

We hence suggest regularization, in particular LASSO regularization, as a valuable methodology to investigate mechanistic changes in KOs. While we do find reduced interactions within the core clock, freeing only core-clock parameters is not sufficient to explain the observed gene expression profiles; for example, the model also requires changes in the ABC transporters to be able to reproduce the KO effects on the network (Fig. 4a).

### Treatment toxicity prediction for HCT116 cell lines

To predict toxicity profiles for irinotecan based on the mRNA expression profile, the model of the transcription-translation network is related to the pharmacokinetics and -dynamics (PK-PD) of irinotecan via the expression of *UGT1A1*, *CES2*, *ABCB* and *ABCC1*.

The protein translation of the mRNAs *UGT1A1*, *CES2*, *ABCB* and *ABCC1*, as well as the free parameters of the PK-PD model part are fitted to cytotoxicity from Hesse et al. 2021^12^, see Methods for details. Protein translation of *UGT1A1*, *CES2*, *ABCB* and *ABCC1* is implemented with circadian protein degradation, as common for many proteins^19^. As in Hesse et al. 2021^12^, maximal protein expression is rescaled to the maximal concentrations used by Dulong et al. 2015^14^. To account for the reduced expression of *UGT1A1* in HCT116 cell lines, 10-fold reduction in the UGT protein expression is assumed for HCT116 cell lines. After this rescaling, UGT is increased in a sigmoidal way following treatment.

Besides the UGT increase, we free the parameters of the PK-PD model part in the equations for the number of living and dead cells, and the cell death modulation. The death rate in Hesse *et al*. 2021^12^ shows an oscillation whose phase is reset by treatment time. The death rate in the model presented here shows a circadian oscillation independent of treatment, plus a transient increase in death rate induced by treatment.

The lack of *UGT1A1* expression in HCT116 cell lines, as well as the here observed changes in other genes relevant for irinotecan metabolism (*CES2*, ABC transporters, see Fig. 2), suggests that the toxicity profile of HCT116 cell lines will likely differ from that of the SW480 cell line.

Using a model fitted to the mRNA expression profile and cytotoxicity data of the SW480 cell line, we modified the mRNA expression profile of the SW480 cell line to the mRNA expression profile of the extended core-clock network fitted to the HCT116 cell lines, see Fig. 2. As the circadian phase evaluated by *ARNTL* expression differs between the cell lines, we adapted the phase of the circadian protein degradation of UGT, CES, ABCB and ABCC, as well as the phase of the circadian modulation in cell death rate to the phase differences observed in *ARNTL*. This allows us to explore how much the toxicity profile is shifted in different clock scenarios, here represented by the various CRC cell lines used. The model predicted that the maximum of the toxicity profile of the HCT116 WT is phase advanced by 1 h compared to the SW480 cell line (Supplementary Fig. 3). Compared to the HCT116 WT, the *PER2*^KO^ showed a phase delay of 1 h, while the *NR1D1*^KO^ and *ARNTL*^KO^ showed toxicity profiles that were phase advanced by about 5 h, see Supplementary Fig. 3c. We observed a slight amplitude reduction in the circadian toxicity profile for HCT116 cell lines. Reduced expression of the irinotecan-inactivating protein UGT1A1 led to overall increased toxicity compared to SW480, visible in Supplementary Fig. 3c as higher Area Under the Curve (AUC) values, as they were normalized to the control without treatment.

### Extrinsic factors -Zeitgebers- can impact the core clock and influence time-dependent toxicity

Zeitgebers are extrinsic factors able to change the circadian rhythm, and thus also the metabolic processes associated with irinotecan action. We used our model to link altered circadian gene expression with the resulting output in the toxicity profile. In particular, we considered short pulses of Zeitgebers as used in clinical therapies, such as administered for light therapy or in the scope of pharmacological interventions^26^.

**Fig. 5:**
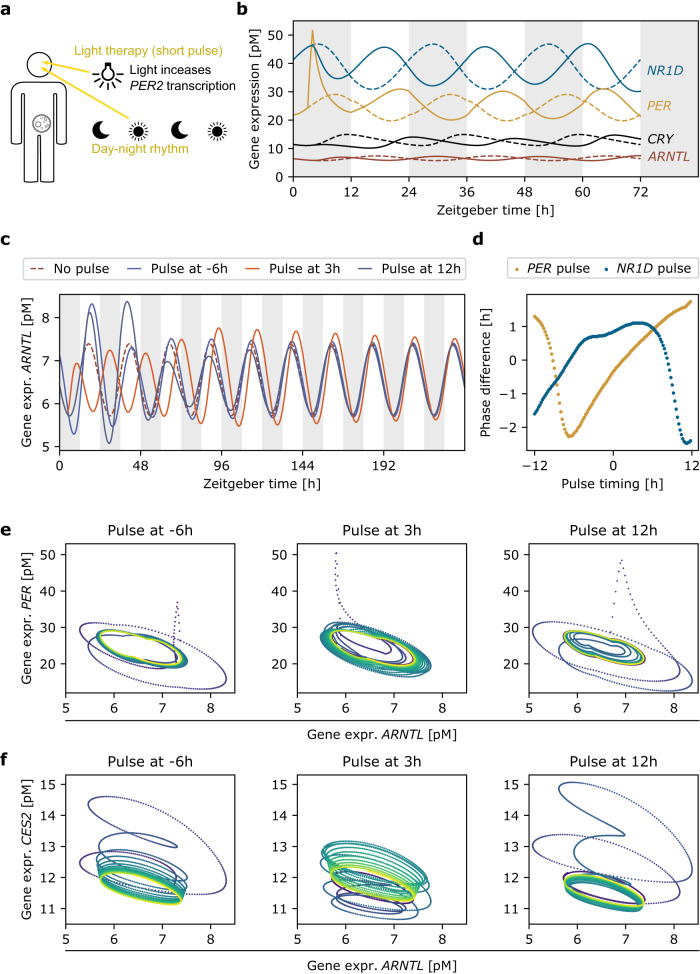
Light induces changes in gene expression, resulting in perturbation-time specific phase differences. **a** The extended core-clock model is subjected to a 24 h light-dark rhythm, via an increase in *PER* transcription rate during light. Light pulses are implemented as stronger increases in *PER* transcription rate. **b** Gene expression of selected core-clock genes in response to a light pulse of 1 h delivered at 3 h zeitgeber time. **c** The response to light pulses depends on the delivery time, with changes in amplitude and phase, here at the example of *ARNTL*. **d** Phase differences observed in *ARNTL* expression in response to pulses in *PER* (mimicking light) or *NR1D* (mimicking pharmacological agents or food) delivered at different time points. **e**, **f** Gene expression oscillation of *PER* (**e**) or *CES2* (**f**) plotted against *ARNTL*. Light pulses increase *PER* (dotted upwards line). Relaxation speed and amplitude changes depend on the time of perturbation and the gene, with *PER* relaxing faster as compared to *CES2*. Strength of the *PER* pulse is 3, except for d where the strength is 1.75, pulse duration is 1 h.

As free-running circadian oscillator, our model showed permanent changes in response to Zeitgeber pulses. To increase similarity with clinical settings, we complemented the model with a 24h-day-night rhythm. This established a reference circadian oscillation, to which the model eventually returns following disturbances. Light exposure is known to increase *PER2* expression both in the SCN and the periphery^27,28^, thus we implemented light in the model as a transient increase in the maximal expression rate of *PER* for the duration of the light exposure, as parameter *f*_light_ in the Supplementary Methods. Our results showed that an increase in the maximal expression rate of *PER* by 7%, i.e. *f*_light_ = 1.07, was sufficient to entrain the circadian oscillator to the day-night rhythm (Fig. 5).

In addition to the day-night Zeitgeber, the model received a short-duration light pulse by transiently increasing *PER* maximal transcription rate. The pulse led to a quick increase in *PER* transcription, which altered the circadian phase of *PER*, and consequently of all other genes (Fig. 5b).

To investigate the impact of pulses, we varied the three parameters defining the pulse, pulse duration, i.e. the duration for which *PER* maximal transcription rate is increased, pulse strength, i.e. the factor *f*_light_ > 1 by which *PER* maximal transcription rate is multiplied, and pulse timing, i.e. the Zeitgeber time point at which *PER* maximal transcription rate is increased. As expected, a longer pulse duration or stronger pulse strength led to larger absolute phase deviations, with a nearly linear correlation in the chosen parameter range (Supplementary Fig. 4). Whether the pulse advanced or delayed the circadian phase depended on the pulse timing (Fig. 5c, d). For an intermediate pulse strength of *f*_light_ = 1.75, possible phase shifts ranged from about −2 h to +2 h (Fig. 5d). Following a strong pulse of *f*_light_ = 3 as in Fig. 5c, the circadian oscillations still relaxed back to the reference oscillations within a couple of days (Fig. 5c). Relaxation speed and changes in oscillation amplitude depended on the pulse timing (Fig. 5e), and differed for different genes (Fig. 5e, f). As expected, core-clock genes, such as *PER*, tend to relax more quickly to the reference oscillation as compared to clock-controlled genes beyond the core clock, such as *CES2* (Fig. 5e, f).

Besides light therapy, also other approaches for shifting circadian phase have been suggested in a cancer context^26^. One possibility may be to change the expression of *NR1D1* by pharmacological agents. We thus evaluated the impact of Zeitgeber pulses in *NR1D* maximal transcription rate on gene expression. For a given pulse strength and duration, pulses in *NR1D* covered a similar range of phase shifts compared to pulses in *PER*, but the timing required for a specific phase shift differed (Fig. 5d). The largest phase shift for *PER* occured at 12 h Zeitgeber time, and for *NR1D* at 3 h Zeitgeber time (Fig. 5d).

**Fig. 6:**
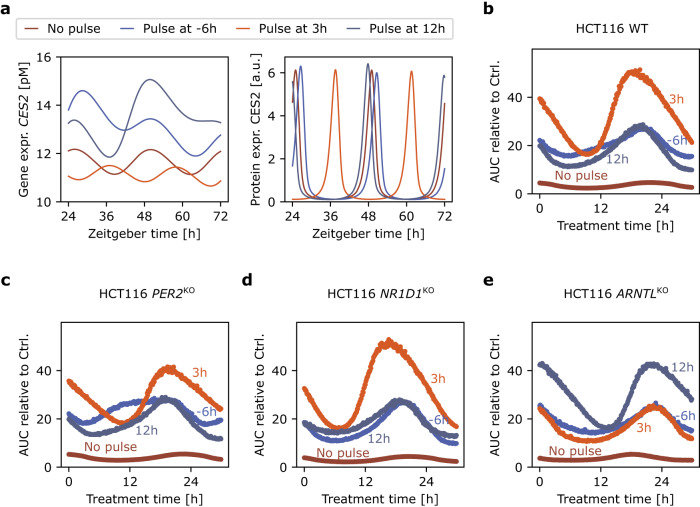
Cytotoxicity profile changes under light therapy. **a** mRNA and protein expression of the irinotecan-activating gene *CES2* for no light pulse, and a 1-h light pulse at −6h, 3 h or 12 h Zeitgeber time for the HCT116 WT. **b** Predicted cytotoxicity profiles for the same light pulses. **c-e** Predicted cytotoxicity profiles for the same light pulses applied to the KO cell lines, HCT116 *PER2*^KO^ (**c**), HCT116 *NR1D1*^KO^ (**d**), and HCT116 *ARNTL*^KO^ (**e**).

The dependence of gene expression on pulse timing also translated to the prediction of toxicity profiles (Fig. 6). The differences in *CES2* gene expression and circadian phase translated into the protein expression (Fig. 6a), and resulted in different timings of maximal toxicity; our example showed differences up to 2 h (Fig. 6b). The timing of the maximal toxicity in response to light pulses depended on the cell line under consideration, with differences observed between HCT116 WT and KO cell lines (Fig. 6b-e), which could potentially represent patients with different internal clock profiles.

Our results showed that the implementation of artificial rhythmic light exposure was able to entrain the phase to a 24 h oscillation. In agreement with our hypothesis that the timing of a perturbation to the clock system is relevant, the sensitivity of our model to pulses showed indeed a dependency on the pulse timepoint. As observed, the pulse strength and duration can additionally scale the effect of the phase shift induced by the chosen pulse timing. For a clinical application, the personalized identification of the appropriate intervention timing is thus essential.

## Discussion

The appropriate timing of chemotherapy can alleviate side-effects, increase efficacy, and thus favour cancer treatment^10^. While previous studies have considered geostationary time to determine the best timepoint of treatment, limited success of chronotherapy might be explained by a lack of personalization of treatment to the endogenous circadian rhythm of the patient. Particularly promising may be the consideration of the patient’s peripheral clock, which in principle mirrors the main pacemaker in the SCN, but is strongly linked to living conditions influenced by behaviour and illness, and can be measured at the molecular level using non-invasive methods^29–33^.

To allow new study designs to consider the state of the peripheral clock, as well as the possible influence of environmental factors, we here investigated the potential for personalization using a transcription-translation network model of circadian gene expression. We simulated personalization of circadian timing using a set of CRC cell lines with different circadian profiles, artificially generated by manipulating their clock. Our results highlight the relevance of compensatory mechanisms in gene expression, paralog compensation, which implies for a set of genes that personalized transcription-translation networks should not be fitted to any individual gene, but rather the sum of the expression of paralogous genes. Paralog compensation has been previously reported also for the human osteosarcoma cell line U-2 OS, and the murine hepatocyte cell line MMH-D3^23,34^. While U-2 OS cells show no paralog compensation for the *PER2*^KO ^^23^, and MMH-D3 cells show an upregulation of *PER3*^34^, we find for HCT116 cells that *PER2* is compensated by *PER1*, not by *PER3*. We find no paralog compensation for *ARNTL*, but rather a reduced expression of *ARNTL2* in the *ARNTL*^KO^, which can be explained by a positive regulation of *ARNTL2* by *ARNTL*, in agreement with previous observations^35^.

We next show that fitting data by a computational model can benefit from regularization methods, in particular LASSO regularization, in order to prevent vastly different parameter sets leading to similar dynamical behaviour. We here used classical LASSO regularization to restrict the parameter divergence relative to the wild type. A potentially more effective alternative for finding essential parameters might be tanh-based error^36^, which is worth considering in subsequent refinements of the model. For personalization of a transcription-translation network, it is necessary to determine a set of reference parameter sets for human tissues, for example by simultaneously fitting gene expression of different humans for one tissue type. Regularization subsequently allows to restrict the parameters to be close to the reference, without the need to reduce the variance in biological realism for the sake of a smaller, and thus better manageable set of parameters. This approach is particularly promising for healthy tissues, while for cancer tissues larger differences in the gene network might be expected between patients.

The used transcription-translation network represents a model of intermediate complexity^32^, the inhibition of PER/CRY on CLOCK/BMAL, for example, is modelled as a blocking of DNA-binding CLOCK/BMAL instead of the combined biological effect of blocking, sequestration, and displacement, as used in another model to enhance robustness of circadian oscillations^37^.

The transcription-translation network connects to a PK-PD model part, which allows to predict the circadian toxicity profile given a certain gene expression profile. As shown previously, the proteins CES2 and UGT1A1, mainly responsible for the activation and subsequent deactivation of irinotecan, have the strongest influence on the circadian toxicity profile of irinotecan treatment^12–14^. This makes HCT116 cells particularly interesting, as we confirm, in accordance with the literature^22^, that under normal conditions, HCT116 cell lines express no *UGT1A1*, nor its paralogs. Interestingly, the toxicity profile of HCT116 predicts a higher toxicity compared to the untreated control than the SW480 cell line, in agreement with the observation that patients with reduced UGT activation show higher sensitivity to irinotecan^38^.

Under certain experimental conditions, HCT116 cells may overexpress UGT1A1, for example, blocking DNA methylation increases *UGT1A1* expression in HCT116 cells^39^. Gene expression of *UGT1A1* is increased during irinotecan/SN38 treatment^20,40^, which motivates the increase in UGT1A1 in the model. Whether or not irinotecan treatment induces *UGT1A1* expression also in HCT116 cells is a question for future research.

Finally, we investigated whether our model could predict the effect of Zeitgebers. This is relevant because therapies that shift circadian phase, such as bright light therapy, have been successfully applied to cancer patients with the aim to minimize side effects like fatigue, most likely via enhancing circadian rhythms^41,42^.

We complemented our model with a 24 h day-night rhythm and tested the effect of pulses in *PER*, representing bright light pulses. Light pulses lead to a transient increase in the SCN, and the information on the light is then transferred to the periphery via multiple channels^43^. Effectively, light pulses induce a shift in the circadian phase, here implemented as an increase in *PER*. Yet, more realistic implementations of light and sensitivity to light might greatly improve the model towards a more clinically usable scenario^44,45^. We found a strong dependence of the resulting phase shift on pulse timing, but also on the specific gene under consideration. This is particularly interesting given the involvement of various circadian genes in chemotherapy-relevant processes. Moreover, we show that pulses in a different gene, *NR1D*, which might result also from the influence of external Zeitgebers like medication or food intake (https://metabolicatlas.org/)^33^, differ in the timing of the maximal phase shifts, making this an attractive alternative if the desired phase shift is not permissive with bright light therapy due to time restrictions. Pulses in *PER* (light) and *NR1D* (pharmacological agents or metabolic processes) led to differential responses of the core clock, suggesting that the reaction of the core clock is specific to the affected gene(s). In the future, we plan to extend our model to explicitly include metabolic dynamics, such as those of the model by Woller et al. 2016^46^. These specific interactions will affect a larger number of core clock genes in response to metabolic signals, and will modulate the altered temporal profile resulting from perturbation induced in core-clock genes. Approaches such as bright light therapy may potentially be used to entrain the peripheral clock of patients to a timepoint where the patient-specific treatment regime overlaps with the ideal treatment timepoint, in particular given that specific treatment hours might be unrealistic due to clinic opening hours.

Our approach to personalization of treatment timing uses a model that relates gene expression with irinotecan toxicity. The model may aid to bridge the observed gap between gene expression and toxicity; for example, mouse data shows only partly a relation between gene expression and toxicity^47^. Promising for irinotecan treatment is also the experimentally observed correlation between *CES2* expression and the activation of irinotecan in tumour tissue^48^.

Optimal treatment timing can be a combination of multiple factors, and dependent on the patient and cancer type, as well as development stage, the best timepoint of treatment could be where the least side effects occur or where the highest cytotoxicity lays, and mathematical models like the one here presented may support clinical decisions in such choices and enable a more personalized treatment planning, for the benefit of the patients.

## Methods

### Cell lines

This study considered data from six different CRC cell lines, RNA-seq data from the wild-type HCT116 cell line and three core-clock knock-outs of the HCT116 cell line (*PER2*^KO^, *NR1D1*^KO^, *ARNTL*^KO^) published in Yalçin et al. 2021^24^ (accession number: E-MTAB-9701), and from SW480 and SW620 (El-Athman et al. 2019^49^, accession number: E-MTAB-7779), as well as for comparison microarray data from SW480 (El-Athman et al. 2018^50^, accession number: E-MTAB-5876).

### Mathematical model

Circadian dynamics in gene expression was modelled by a transcription-translation network including core-clock genes and core-clock regulated genes relevant for irinotecan metabolism. This model was fitted to mRNA expression derived from RNA-seq data for six different cell lines, the wild-type HCT116 cell line, three core-clock knock-outs of the HCT116 cell line (*PER2*^KO^, *NR1D1*^KO^, *ARNTL*^KO^) and for comparison with the previous model the SW480 cell line and the SW620 cell line. For the SW480 cell line, the model is for comparison also fitted to microarray data, which was rescaled to the same mean as the RNA-seq data.

The PK-PD model from Hesse et al. 2021^12^ is refined in the *UGT1A1* expression and a circadian modulation of the cell death rate. After rescaling to the appropriate protein concentration, see Supplementary Methods, *UGT* is increased following treatment in a sigmoidal way. The increase in UGT is modelled by multiplying the protein expression resulting from the translation step and the rescaling by a sigmoidal curve *sig*(*t*) with free magnitude *M*_UGT_ and free slope *k*_UGT_,

1 $$\begin{array}{c}{{{sig}}}\left(t\right)=1+\frac{{M}_{{\rm{UGT}}}}{1+{\rm{exp }}\left({k}_{{\rm{UGT}}}t+4\right)}\end{array}$$

with *t* as time after treatment.

The death rate in the model presented here shows a circadian oscillation, plus a transient increase in death rate, which is modelled by an alpha function, see Supplementary Methods.

### Model fitting of the transcription-translation network

Parameter optimization used the evolutionary algorithm CMA-ES^51^ via the Python implementation pycma on a compute cluster. The cost function is the squared error between data and model fit, evaluating for each mRNA *j* the simulation *s*^*j*^(*t*) at the same time points *t*_*i*_ as the data *x*^*j*^_*i*_ was sampled, normalized by the maximum gene expression of the mRNA expression data, summed for all genes in the network:

2 $${cost}_{{\rm{SE}}}=\sum _{i,j}{\left(\frac{{x}_{i}^{j}-{s}^{j}\left({t}_{i}\right)}{\mathop{\max }\nolimits_{k}{x}_{k}^{j}}\right)}^{2}$$

Here the maximum is taken for each mRNA over the experimental time series. The division ensures equal weight to all mRNAs independent of their concentration.

For the numerical integration we used Python’s scipy.integrate.odeint (method: lsoda, relative tolerance = 10^−4^, absolute tolerance = 10^−12^). Model fits of mRNA were forced to oscillate with a minimum relative amplitude of 5%, i.e. for the maximum and minimum of the simulated time series we demanded (max-min)/max > 0.05, with the exception of *UGT1A1* for the HCT116 cell lines, which was not expressed in these cell lines.

### Regularization of the model fit

For LASSO regularization, the cost based on the squared error between data and simulation from above was extended by the following penalty on the parameters:

3 $${cost}_{{{\rm{LASSO}}}}= {cost}_{{{\rm{SE}}}}+\lambda \frac{1}{{n}_{{{\rm{par}}}}}\sum _{i}{\rm{abs}}\left(\frac{{p}_{i}-{p}_{i}^{{{\rm{WT}}}}}{{p}_{i}^{{{\rm{WT}}}}}\right)$$

where abs() is the absolute value, *p*_i_ is the *i*th fitted parameter, ${n}_{{\rm{par}}}$ the number of parameters, and *p*_i_^WT^ is the *i*th parameter from the fit of the wild type. The parameter *λ* is named penalty term, and the larger this term, the more parameters are forced to remain close to the wild-type parameter set. The penalty term *λ* was chosen by evaluating for different *λ* the squared error and the number of parameters with at least 5% deviation from the wild-type parameters, see Fig. 4b, c. We have chosen *λ* with a squared error comparable to the case without penalty, but with a clearly reduced number of parameters with a large deviation.

### Circadian toxicity profiles

The experimental circadian toxicity profile results from a calculation of the AUC for the experimental cytotoxicity curve shown in Supplementary Fig. 3a as triangles. Cytotoxicity is the experimental measure of the abundance of dead cells; it counts red florescent objects which result from the binding of a cytox dye to dead cells, see Hesse et al. 2021 for experimental details^12^. The cytotoxicity curves are first rescaled to start at the same value as the control condition also for treated conditions, to ensure that the toxicity profile depends on the temporal development of the cytotoxicity, but not the initial values at treatment onset.

For a model definition of the PK-PD part see Supplementary Methods. The dynamical variable for the number of dead cells *D* (see Supplementary Methods) is fitted to the experimental cytotoxicity curves of the SW480 cell line, see Supplementary Fig. 3a. A calculation of the AUC for this dynamic variable *D* gives us the simulated circadian toxicity profile.

As for the transcription-translation network, parameter optimization used the evolutionary algorithm CMA-ES via the Python implementation pycma on a compute cluster. The cost function is the squared error between cytotoxicity data and model fit. For the numerical integration we used Python’s scipy.integrate.solve_ivp (method: lsoda, relative tolerance = 10^−4^, absolute tolerance between 10^−3^ and 10^−7^).

### Modulation of mRNA expression by light

Zeitgebers, such as bright light pulses, can shift circadian rhythms. We asked whether a simple implementation of light as a transient increase in the dynamical variable *PER* (modelling the sum of *PER1*, *PER2* and *PER3*) can shift the toxicity profile of the model simulation. Light was implemented as a change in the maximal transcription rate of *PER*, i.e. the light-sensitive transcription rate *V1max*_light_ is given as

4 $${{V{\it{1}}max}}_{{{\mathrm{light}}}}=\left\{\begin{array}{cc}{{V{\it{1}}max}} , & {\rm{if}}\; {\rm{light}}\; {\rm{off}}\\ f\,{{V{\it{1}}max}}, & {\rm{if}}\; {\rm{light}}\; {\rm{on}}\end{array}\right.$$

where *V1max* is the maximal transcription rate of *Per* without light, and *f* > 1 is a constant factor accounting for the increase in transcription rate with light on, see Supplementary Methods.

The strength of the rhythmic light entrainment was determined by the period of a harmonic oscillation of 24 h and a relative amplitude over 64 h that is at least similarly strong as without light.

We investigated the response of the system in response to a short and strong light pulse with a duration of *dur*_pulse_ = 1 h and a strength of *f*_light_ = 3 unless stated otherwise, given at a certain perturbation time *T*_pulse_. If the transcription-translation network was not entrained, light pulse stimulation induced persistent shifts in the phase of the simulated mRNA expression. For a more realistic setting, we entrained the circadian oscillations of the model with a zeitgeber light that follows a 12 h dark-12h light cycle, with a strength of *f*_light_ = 1.07. Giving a light pulse stimulus in the entrained system led to a transient shift in the phase of the mRNA oscillations.

We evaluated the phase shift induced by light pulses with different parameters on the gene expression of *ARNTL*. The relative phase shift was measured as the time difference of the maximum of *ARNTL* in the second maximum after the pulse comparing the condition with light pulse with the condition without light pulse.

We also tested the effect of pulses that increase the expression of *NR1D*, implemented in analogue to the pulses on *PER*.

### Reporting summary

Further information on research design is available in the Nature Research Reporting Summary linked to this article.

## Supplementary information

Supplemental material

Reporting Summary

## Data Availability

The datasets analysed during the current study are available as follows: recently generated RNA-sequencing data for HCT116 WT cells and KOs in this study have been deposited in the ArrayExpress repository (Accession number: E-MTAB-9701). Other data sets used in this manuscript are available as follows: SW480 and SW620 cell lines, ArrayExpress, E-MTAB-7779 and E-MTAB-5876.

## Code availability

The code for the mathematical model described in this work is available at bioModels under the identifier MODEL2305270001.
