# Supplementary material for: An integrative mathematical model for timing treatment toxicity and Zeitgeber impact in colorectal cancer cells
Source: NPJ Syst Biol Appl. 2023 Jun 23;9:27. doi: 10.1038/s41540-023-00287-4 (PMC10290159; doi:10.1038/s41540-023-00287-4)
Supplement: Supplementary file 1 — Supplemental material [file 41540_2023_287_MOESM1_ESM.pdf]

Supplementary Material

An integrative mathematical model for timing treatment toxicity and Zeitgeber impact in colorectal cancer cells

Janina Hesse[1], Tim Müller[2], Angela Relógio[1,2,3*]

[1]Institute for Systems Medicine, Faculty of Human Medicine, MSH Medical School Hamburg, Hamburg 20457, Germany
[2]Institute for Theoretical Biology (ITB), Charité – Universitätsmedizin Berlin, corporate member of Freie Universität Berlin, Humboldt-Universität zu Berlin, and Berlin Institute of Health, Berlin 10117, Germany
[3]Molecular Cancer Research Center (MKFZ), Medical Department of Hematology, Oncology, and Tumor Immunology, Charité – Universitätsmedizin Berlin, corporate member of Freie Universität Berlin, Humboldt-Universität zu Berlin, and Berlin Institute of Health, Berlin 10117, Germany

[*]Corresponding author: angela.relogio@medicalschool-hamburg.de

Supplementary figures

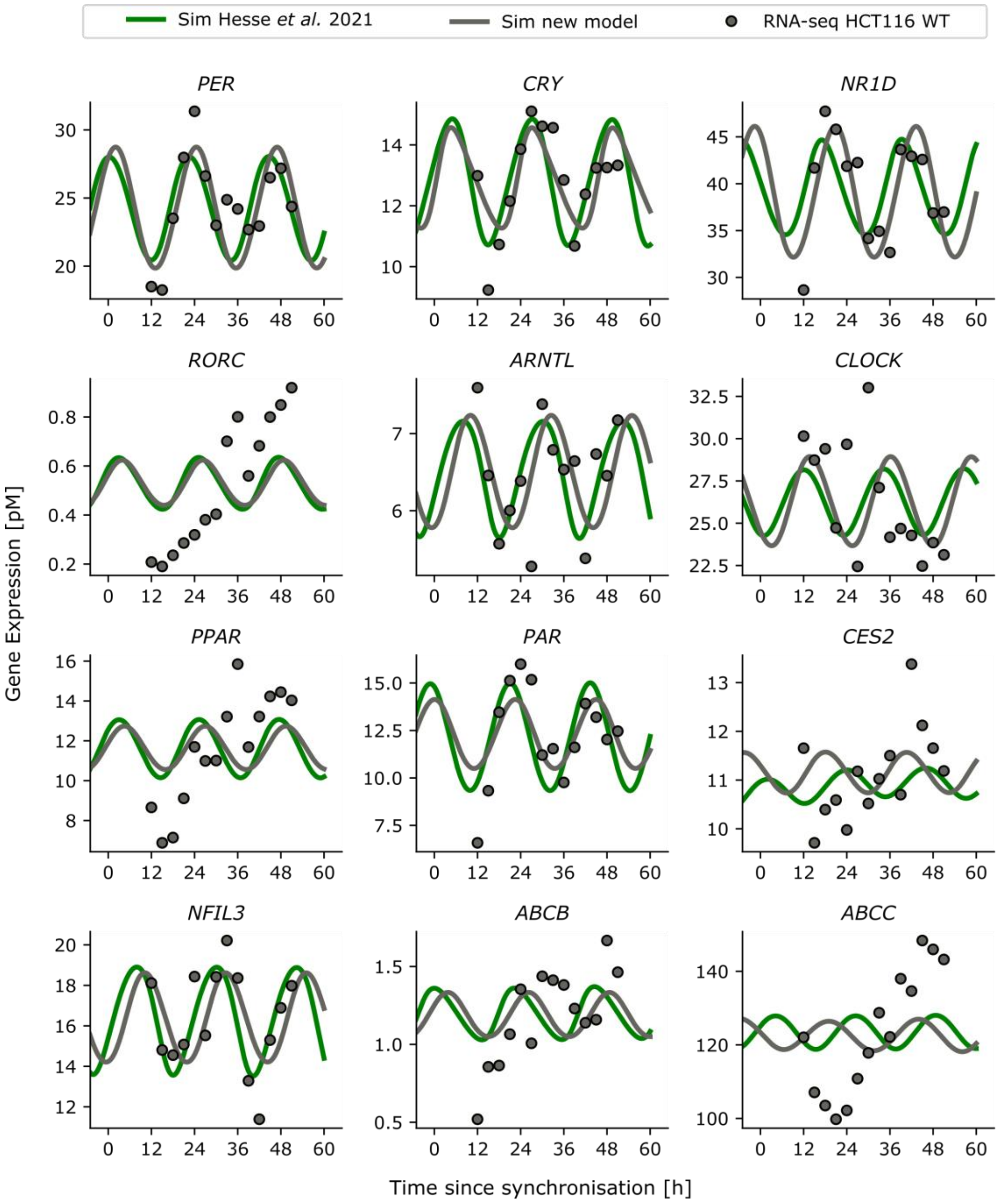

Supplementary Figure 1: Comparison of transcription-translation network models. Fit of the new model to the HCT116 WT data (grey line and dots, R^2 value of 0.29) improve the fit that uses the model as stated in Hesse *et al*. 2021 [3] (green line, R^2 value of 0.23). The improvement results in particular from a better fit of *CES2* and *ABCC*.

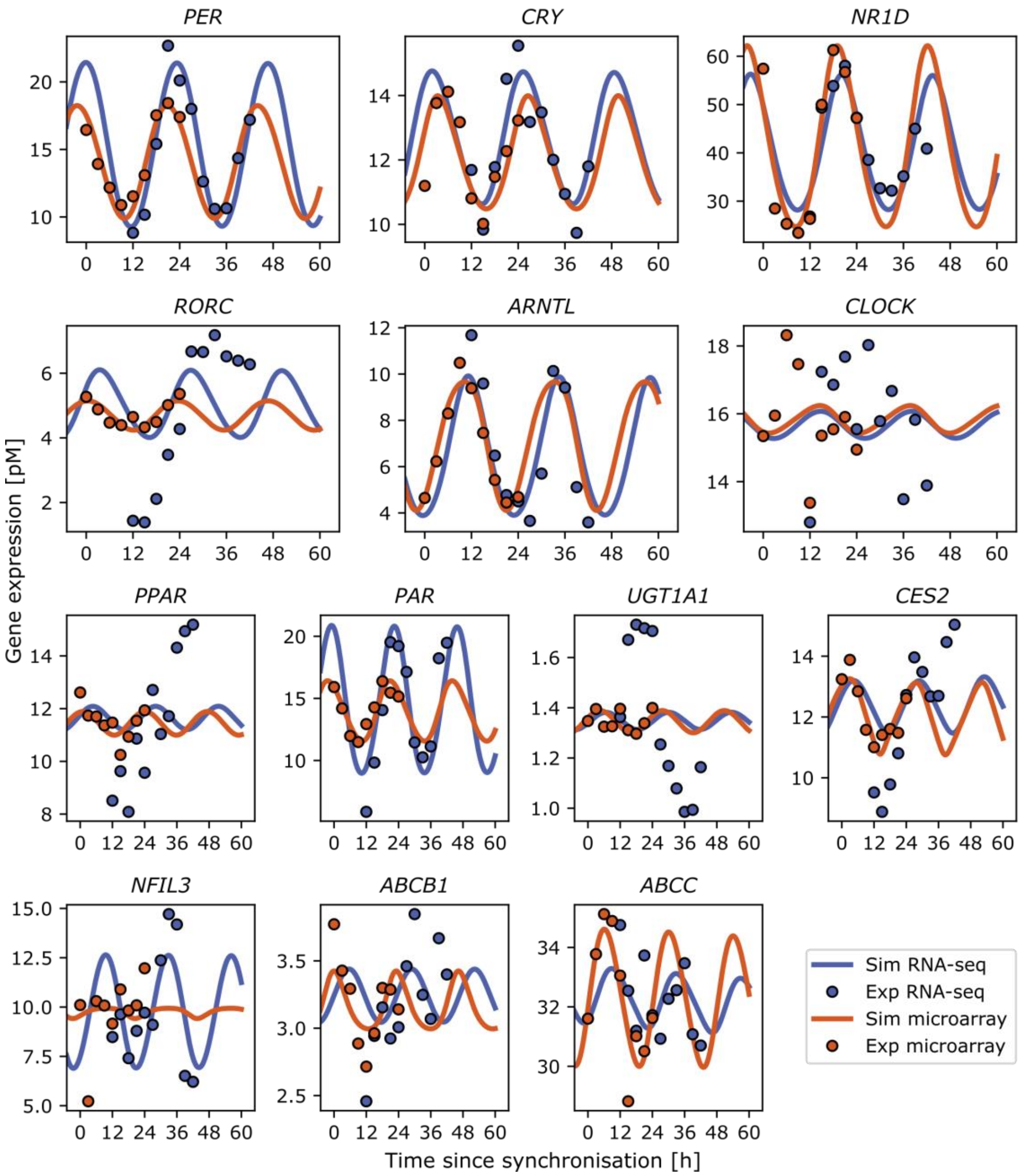

Supplementary Figure 2: Model fit to SW480 cell line. For the SW480 cell line, RNA-seq data and microarray data was fitted. The RNA-seq data (blue circles) was sampled over a longer time (30h versus 24h) than the microarray data (green circles). Genes with a linear trend in the RNA-seq data (blue lines) are still fitted in reasonable agreement with the microarray data (green lines). Microarray data was rescaled to the same mean as the RNA-seq data.

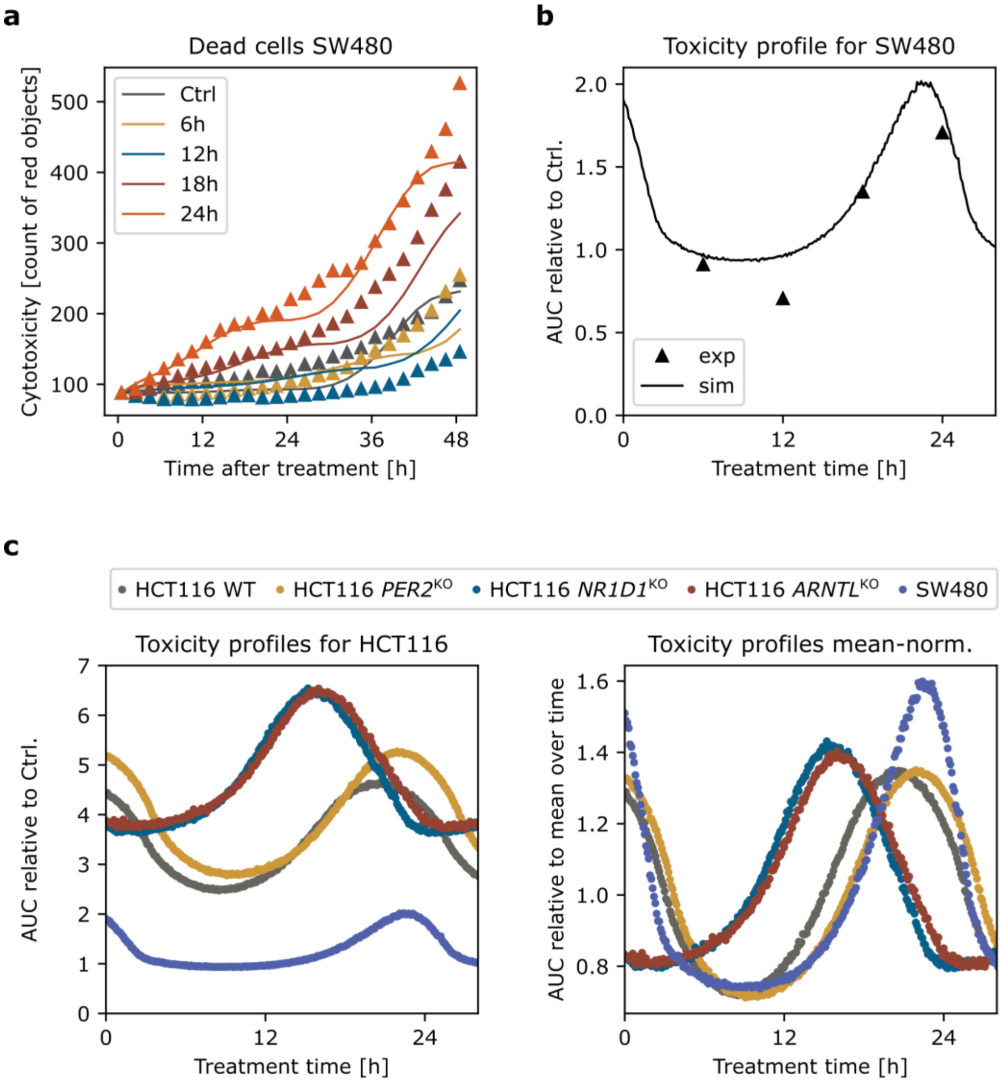

Supplementary Figure 3: Circadian toxicity profiles. a Cytotoxicity of the SW480 cell line depends on the timing of the treatment. Strong diurnal variation in the experimental data (triangles) can be reproduced by a model (straight line). **b** The toxicity profile is plotted as the Area Under the Curve (AUC) of the cytotoxicity curves, normalized by the control condition, against treatment time for experimental (exp) and simulated (sim) data. **c** Using the same model with the gene expression of the HCT116 cell lines as input, the circadian toxicity profile changes (left). The same data as in the left panel is shown in the right panel mean-normalized to compare relative amplitudes and phases.

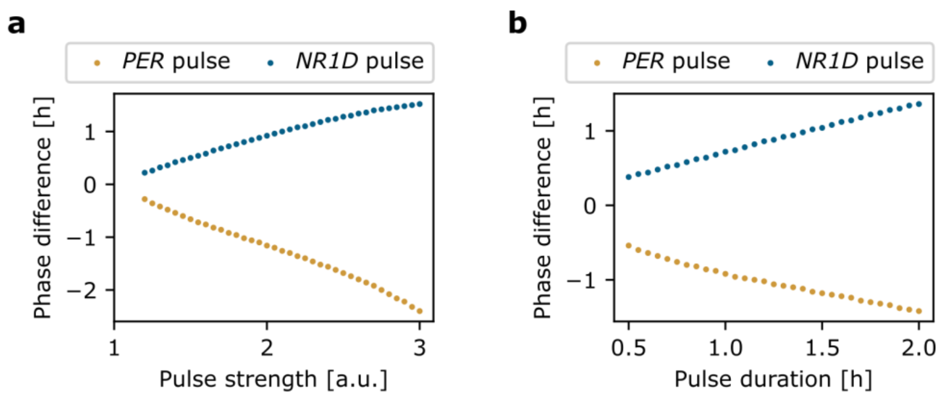

Supplementary Figure 4: Pulse strength and duration modulate the phase difference of model simulations with and without pulse. An increase in pulse strength (a) or duration (b) can be used to strengthen the phase advance or delay for each stimulation time point.

Supplementary Methods

Ordinary differential equation model for the transcription-translation network with 34 dynamic variables, see **Supplementary Table 1**. Refined model based on the model published in Hesse *et al.* 2021 [1], differences to this model are marked in blue font colour. Model parameters for the extended core-clock model fitted to the different cell lines are stated in **Supplementary Table 2**.

Supplementary Table 1: Dynamic variables of the extended core-clock model.

Variable name	Species name
x_1	CLOCK/BMAL$_N$
x_2	PER/CRY$_N^{tot}$
x_5	REV-ERB$_N$
REV^*	activated REV-ERB$_N$
x_6	ROR$_N$
y_1	*PER*
y_2	*CRY*
y_3	*NR1D*
y_4	*ROR*
y_5	*ARNTL*
y_6	*CLOCK*
z_1	CRY$_C$
z_2	PER$_C^{tot}$
z_4	PER/CRY$_C^{tot}$
z_5	CLOCK$_C$
z_6	NR1D$_C$
z_7	ROR$_C$
z_8	ARNTL$_C$
z_9	CLOCK/BMAL$_C$
Ces	*CES2*
Ugt	*UGT1A1*
$Abcb$	*ABCB1*
$Abcc$	*ABCC*
$Ppar$	*PPARα*
Par	*PAR bZip*
$Nfil$	*NFIL3*
CES	CES2

UGT	UGT1A1
$ABCB$	ABCB1
$ABCC$	ABCC
$PPAR$	PPARα
PAP	PAR bZIP
$NFIL$	NFIL3
$NFIL^*$	activated NFIL3

Model equations

Core-clock genes and proteins

CLOCK/BMAL$_N$

$$\frac{dx_1}{dt} = k_{i_{z_9}} z_9 - k_{e_{x_1}} x_1 - d_{x_1} x_1 \quad (1)$$

PER/CRY$_N^{tot}$

$$\frac{dx_2}{dt} = k_{i_{z_4}} z_4 - k_{e_{x_2}} x_2 - d_{x_2} x_2 \quad (2)$$

REV-ERB$_N$

$$\frac{dx_5}{dt} = k_{i_{z_6}} z_6 - d_{x_5} x_5 \quad (3)$$

REV-ERB activated protein with circadian protein degradation with a 24h period:

$$\frac{d[REV^*]}{dt} = r_{t_{\mathrm{REV}}} x_5 - d_{\mathrm{REV}} \left(1 + A_{\mathrm{REV}} \cos\left(\frac{2\pi}{24h} t + \phi_{\mathrm{REV}}\right)\right) [REV^*] \quad (4)$$

ROR$_N$

$$\frac{dx_6}{dt} = k_{i_{z_7}} z_7 - d_{x_6} x_6 \quad (5)$$

Per

$$\frac{dy_1}{dt} = f_{\text{light}} V_{1_{\text{max}}} \frac{1 + a\left(\frac{x_1}{k_{t_1}}\right)^b}{1 + \left(\frac{x_2}{k_{i_1}}\right)^c \left(\frac{x_1}{k_{t_1}}\right)^b + \left(\frac{x_1}{k_{t_1}}\right)^b} \frac{1}{1 + \left(\frac{[\text{NFIL}]}{i_{Per}}\right)^c} - d_{y_1} y_1 \qquad (6)$$

Cry

$$\frac{dy_2}{dt} = V_{2_{\text{max}}} \frac{1 + d\left(\frac{x_1}{k_{t_2}}\right)^e}{1 + \left(\frac{x_2}{k_{i_2}}\right)^f \left(\frac{x_1}{k_{t_2}}\right)^e + \left(\frac{x_1}{k_{t_2}}\right)^e} \frac{1}{1 + \left(\frac{x_5}{k_{i_{21}}}\right)^{f_1}} - d_{y_2} y_2 \qquad (7)$$

Rev-Erb

$$\frac{\text{d}y_3}{\text{d}t} = f_{\text{pharma}} V_{3\text{max}} \frac{1 + g\left(\frac{x_1}{k_{t_3}}\right)^b}{1 + \left(\frac{x_2}{k_{i_4}}\right)^c \left(\frac{x_1}{k_{t_3}}\right)^b + \left(\frac{x_1}{k_{t_3}}\right)^b} \frac{1}{1 + \left(\frac{[\text{NFIL}]}{i_{\text{Rev}}}\right)^c} - d_{y_3} y_3 \qquad (8)$$

Ror

$$\frac{dy_4}{dt} = V_{4_{\text{max}}} \frac{1 + h\left(\frac{x_1}{k_{t_4}}\right)^b}{1 + \left(\frac{x_2}{k_{i_4}}\right)^c \left(\frac{x_1}{k_{t_4}}\right)^b + \left(\frac{x_1}{k_{t_4}}\right)^b} - d_{y_4} y_4 \qquad (9)$$

ARNTL

$$\frac{dy_5}{dt} = V_{5_{\text{max}}} \frac{1 + i\left(\frac{x_6}{k_{t_5}}\right)^b}{1 + \left(\frac{x_5}{k_{i_5}}\right)^c + \left(\frac{x_6}{k_{t_5}}\right)^b} - d_{y_5} y_5 \qquad (10)$$

Clock

$$\frac{dy_6}{dt} = V_{6_{\max}} \frac{1 + j\left(\frac{x_6}{k_{t_6}}\right)^b}{1 + \left(\frac{x_5}{k_{i_6}}\right)^c + \left(\frac{x_6}{k_{t_6}}\right)^b} - d_{y_6} y_6 \tag{11}$$

CRY_C

$$\frac{dz_1}{dt} = k_{p_2} y_2 + k_{d_{z_4}} z_4 - k_{f_{z_4}} z_1 z_2 - d_{z_1} z_1 \tag{12}$$

PER_C^{tot}

$$\frac{dz_2}{dt} = k_{p_1} y_1 + k_{d_{z_4}} z_4 - k_{f_{z_4}} z_1 z_2 - d_{z_2} z_2 \tag{13}$$

PER/CRY_C^{tot}

$$\frac{dz_4}{dt} = k_{f_{z_4}} z_1 z_2 + \frac{v_c}{v_n} k_{e_{x_2}} x_2 - \frac{v_c}{v_n} k_{i_{z_4}} z_4 - k_{d_{z_4}} z_4 \tag{14}$$

CLOCK_C

$$\frac{dz_5}{dt} = k_{p_6} y_6 + k_{d_{z_9}} z_9 - k_{f_{z_9}} z_8 z_5 - d_{z_5} z_5 \tag{15}$$

REV-ERB_C

$$\frac{dz_6}{dt} = k_{p_3} y_3 - \frac{v_c}{v_n} k_{i_{z_6}} z_6 - d_{z_6} z_6 \tag{16}$$

ROR_C

$$\frac{dz_7}{dt} = k_{p_4} y_4 - \frac{v_c}{v_n} k_{i_{z_7}} z_7 - d_{z_7} z_7 \tag{17}$$

ARNTL_C

$$\frac{dz_8}{dt} = k_{p_5} y_5 + k_{d_{z_9}} z_9 - k_{f_{z_9}} z_8 z_5 - d_{z_8} z_8 \tag{18}$$

CLOCK/BMAL_C

$$\frac{dz_9}{dt} = k_{f_{z_9}} z_8 z_5 + \frac{v_c}{v_n} k_{e_{x_1}} x_1 - \frac{v_c}{v_n} k_{i_{z_9}} z_9 - k_{d_{z_9}} z_9 \tag{19}$$

Clock-controlled proteins

For PPARα, PAR-bZip, UGT1A1, CES2, NFIL3, ABCB1 and ABCC, the dynamics for translation and where relevant import into the nucleus has the same structure for each $PROTEIN$:

$$\frac{\mathrm{d}PROTEIN}{\mathrm{d}t} = r_{\mathrm{PROTEIN}}\, mRNA - d_{\mathrm{PROTEIN}}\, PROTEIN \tag{20}$$

The degradation rate d_{PROTEIN} is a constant for PPARα, PAR-bZip, and NFIL3. For UGT1A1, CES2, ABCB1 and ABCC, the transciption rate $r_{\mathrm{PROTEIN}} = 1$, as this parameter only scales protein abundances (which are rescaled at a later stage, see Supplementary Methods, section Pharmacokinetics and -dynamics), and the degradation rate follows a circadian rhythm,

$$d_{\mathrm{PROTEIN}}(t) = \gamma_{\mathrm{PROTEIN}}\big(1 + A_{\mathrm{PROTEIN}} \cos(\omega t + \phi_{\mathrm{PROTEIN}})\big), \tag{21}$$

with $\omega = \frac{2\pi}{T}$, where T is the period of the circadian oscillation of the mRNA, evaluated based on the simulated gene expression.

Similar to NR1D, also for NFIL3 we introduce an activated protein with circadian protein degradation with a 24h period:

$$\frac{d[NFIL^*]}{dt} = r_{t_{\mathrm{NFIL}^*}} [NFIL] - d_{\mathrm{NFIL}^*} \left(1 + A_{\mathrm{NFIL}} \cos\left(\frac{2\pi}{24h} t + \phi_{\mathrm{NFIL}}\right)\right) [NFIL^*] \tag{22}$$

Clock-controlled mRNAs

For *PPARα, PAR-bZip, UGT1A1, CES2, NFIL3, ABCB1* and *ABCC*, the dynamics for transcription has the same structure for each $mRNA$:

$$\frac{\mathrm{d}mRNA}{\mathrm{d}t} = V_{\mathrm{mRNA}} \mathbb{T}(mRNA) - d_{\mathrm{mRNA}}\, mRNA, \tag{23}$$

with the following transcription functions.

Pparα

$$\mathbb{T}(Ppar) = \frac{1 + f_{\mathrm{Ppar}} \left(\frac{x_1}{a_{\mathrm{Ppar}}}\right)^b}{1 + \left(\frac{x_2}{i_{\mathrm{Ppar}}}\right)^c \left(\frac{x_1}{a_{\mathrm{Ppar}}}\right)^b + \left(\frac{x_1}{a_{\mathrm{Ppar}}}\right)^b} \tag{24}$$

PAR bZip

$$\mathbb{T}(Par) = \frac{1 + f_{\mathrm{Par}} \left(\frac{x_1}{a_{\mathrm{Par}}}\right)^b}{1 + \left(\frac{x_2}{i_{\mathrm{Par}}}\right)^c \left(\frac{x_1}{a_{\mathrm{Par}}}\right)^b + \left(\frac{x_1}{a_{\mathrm{Par}}}\right)^b} \tag{25}$$

Ugt1a1

$$\mathbb{T}(Ugt) = \frac{1 + f_{\mathrm{Ugt}} \left(\frac{[\mathrm{PPAR}]}{a_{\mathrm{Ugt}}}\right)^b}{1 + \left(\frac{[\mathrm{PPAR}]}{a_{\mathrm{Ugt}}}\right)^b} \tag{26}$$

Nfil3

$$\mathbb{T}(Nfil) = \frac{1 + f_{\mathrm{Nfil}} \left(\frac{x_6}{a_{\mathrm{Nfil}}}\right)^b}{1 + \left(\frac{x_5}{i_{\mathrm{Nfil}}}\right)^c + \left(\frac{x_6}{a_{\mathrm{Nfil}}}\right)^b} \tag{27}$$

Ces

$$\mathbb{T}(Ces) = \frac{1 + f_{\text{Ces}} \left(\frac{1e-11}{a_{\text{Ces}}}\right)^b}{1 + \left(\frac{[REV^*]}{i_{\text{Ces}}}\right)^c + \left(\frac{1e-11}{a_{\text{Ces}}}\right)^b} \frac{1}{1 + \left(\frac{[PPAR]}{i_{\text{CesPPar}}}\right)^c} \tag{28}$$

a_{Ces} is the inhibition strength of unspecified constantly expressed proteins.

Abcb1

$$\mathbb{T}(Abcb) = \frac{1 + f_{\text{Abcb}} \left(\frac{[\text{PAR}]}{a_{\text{Abcb}}}\right)^b}{1 + \left(\frac{[\text{NFIL}]}{i_{\text{Abcb}}}\right)^c \left(\frac{[\text{PAR}]}{a_{\text{Abcb}}}\right)^b + \left(\frac{[\text{PAR}]}{a_{\text{Abcb}}}\right)^b} \tag{29}$$

Abcc

$$\mathbb{T}(Abcc) = \frac{1 + f_{\text{Abcc}} \left(\frac{[\text{PAR}]}{a_{\text{Abcc}}}\right)^b}{1 + \left(\frac{[\text{NFIL}^*]}{i_{\text{Abcc}}}\right)^c \left(\frac{[\text{PAR}}{a_{\text{Abcc}}}\right)^b + \left(\frac{[\text{PAR}}{a_{\text{Abcc}}}\right)^b} \tag{30}$$

Pharmacokinetics and -dynamics

The protein expression of UGT1A1, CES2, ABCB1, and ABCC is rescaled to a maximal expression of 1361.424, 0.78064, 17.16708, and 1096.277, respectively. For simulation if the HCT116 cell lines, UGT1A1 expression is reduced to 10%.

The time t is in the following the time since treatment. Treatment starts at T_{treat} in Zeitgeber time (the time since synchronization if no explicit light Zeitgeber is included).

$$\frac{d[CPT_{out}]}{dt} = \frac{V_{in}}{V_{out}\left(-k_{upCPT}[CPT_{out}] + \frac{V_{effCPT}[ABCB][CPT_{in}]}{K_{effCPT} + [CPT_{in}]}\right)} \tag{31}$$

$$\frac{d[CPT_{in}]}{dt} = k_{upCPT}[CPT_{out}] - \frac{V_{effCPT}[ABCB][CPT_{in}]}{K_{effCPT} + [CPT_{in}]} - \frac{V_{ces}[CES][CPT11_{in}]}{K_{ces} + [CPT11_{in}]} \tag{32}$$

$$\frac{d[SN_{out}]}{dt} = \frac{V_{in}}{V_{out}\left(-k_{upSN}[SN_{out}] + \frac{V_{effSN}[ABCC][SN_{in}]}{K_{effSN} + [SN_{in}]}\right)} \tag{33}$$

$$d\frac{[SN_{in}]}{dt} = k_{upSN}[SN_{out}] - \frac{V_{effSN}[ABCC][SN_{in}]}{K_{effSN} + [SN_{in}]} + \frac{V_{ces}[CES][CPT_{in}]}{K_{ces} + [CPT_{in}]} - \frac{V_{ugt}[UGT][SN_{in}]}{K_{ugt} + [SN_{in}]} - k_{f2}[DNATOP1][SN_{in}] + k_{r2}[Compl] \tag{34}$$

$$\frac{d[TOP1]}{dt} = k_{ftop} - k_{dtop}[TOP] - k_{f1}[TOP1]\left[DNA_{free}\right] + k_{r1}[DNATOP1] + k_{r2}[Compl] \tag{35}$$

$$\frac{d[DNATOP1]}{dt} = k_{f1}[TOP]\left[DNA_{free}\right] - k_{f2}[DNATOP1][SN_{in}] - k_{r1}[DNATOP1] \tag{36}$$

$$\frac{d[Compl]}{dt} = k_{f2}[DNATOP1][SN38_{in}] - k_{r2}[Compl] - k_{Irr}[Compl] \tag{37}$$

$$\frac{d[Icompl]}{dt} = k_{Irr}[Compl] \tag{38}$$

The death rate is given by a circadian modulation in death rate, and the toxicity of irinotecan, which is transiently increased by treatment in form of an alpha function:

$$d_r(t) = \left(k_{\text{apop}}\left(A_{\text{alpha}}\frac{t}{\tau}\exp\left(1 - \frac{t}{\tau}\right) + 1\right)([Compl] + [Icompl]) + k_{\text{control}}\right)\left(1 + A_{\text{apop}}\cos\left(\omega(t + T_{\text{treat}}) + \phi_{\text{apop}}\right)\right) \tag{39}$$

ω is $2\pi/T$, where T is the period of the circadian mRNA as defined above in section Clock-controlled proteins.

Number of living cells, N:

$$\frac{\mathrm{d}N}{\mathrm{d}t} = \left(k_{\text{prol}} - p_{\text{treat}}\right)N - d_r N \tag{40}$$

For the untreated case, $p_{\text{treat}} = 0$, and for the treated cases, $p_{\text{treat}} = const. > 0$.

Number of dead cells, D:

$$\frac{\mathrm{d}D}{\mathrm{d}t} = d_r N \tag{41}$$

Supplementary References

1 Hesse, J., Martinelli, J., Aboumanify, O., Ballesta, A. & Relogio, A. A mathematical model of the circadian clock and drug pharmacology to optimize irinotecan administration timing in colorectal cancer. *Comput Struct Biotechnol J* **19**, 5170-5183, doi:10.1016/j.csbj.2021.08.051 (2021).

Supplementary Table 2: Parameters of the extended core-clock model. *The model parameters correspond to the model fit with LASSO regularization for the HCT116 KO cell lines, and the fit without regularization for HCT116 WT, SW480 and SW620.*

Parameter	Name	HCT116 WT	HCT116 PER2KO	HCT116 NR1D1KO	HCT116 ARNTLKO	SW480	SW620
Degradation rates for nuclear proteins or nuclear protein complexes [hour^{-1}]							
$\boldsymbol{d_{x_1}}$	CLOCK/BMAL	0.924	0.962	1.04	0.923	0.988	0.202
$\boldsymbol{d_{x_2}}$	PER/CRY$_N^{tot}$	0.0787	0.0769	0.0857	0.0787	0.0784	0.0787
$\boldsymbol{d_{x_5}}$	REV-ERB$_N$	2.23	2.38	2.55	2.19	2.09	2.35
$\boldsymbol{d_{x_6}}$	ROR$_N$	2.15	2.24	2.16	2.42	2.15	2.36
Degradation rates for mRNAs [hour^{-1}]							
$\boldsymbol{d_{y_1}}$	*Per*	0.675	0.673	0.695	0.789	0.768	0.975
$\boldsymbol{d_{y_2}}$	*Cry*	0.0262	0.0233	0.0275	0.027	0.0296	0.021
$\boldsymbol{d_{y_3}}$	*Rev-Erb*	2.31	2.32	2.27	2.32	2.26	1.67
$\boldsymbol{d_{y_4}}$	*Ror*	0.183	0.183	0.182	0.154	0.147	0.124
$\boldsymbol{d_{y_5}}$	*Bmal*	2.83	2.84	2.81	2.83	2.63	2.74
$\boldsymbol{d_{y_6}}$	*Clock*	0.125	0.127	0.125	0.126	0.076	0.0451

Degradation rates for cytoplasmic proteins [hour^{-1}]							
d_{z_1}	CRY_C	0.931	0.93	1.01	0.879	0.95	0.973
d_{z_2}	PER_C	3.0	2.98	2.96	2.96	2.68	2.98
d_{z_5}	CLOCK_C	2.85	2.89	2.81	2.85	3.0	2.75
d_{z_6}	REV-ERB_C	3.0	2.91	2.9	3.0	2.97	2.7
d_{z_7}	ROR_C	0.016	0.0188	0.0167	0.0148	0.0151	0.0166
d_{z_8}	BMAL_C	2.07	2.43	2.29	2.04	2.08	2.04
Reaction rates for complex formation [mol× L^{-1} ×hours^{-1}]							
$k_{f_{z_9}}$	CLOCK/BMAL_C	9.18e+07	9.2e+07	5.33e+07	9.19e+07	9.15e+7	1.09e+8
$k_{f_{z_4}}$	PER/CRY_C^{tot}	23.5	23.4	23.9	14.8	21.4	23.4
Reaction rates for complex dissociation [hours^{-1}]							
$k_{d_{z_9}}$	CLOCK/BMAL_C	7.28e+06	7.27e+06	7.29e+06	7.28e+06	7.28e+6	6.27e+6
$k_{d_{z_4}}$	PER/CRY_C^{tot}	0.704	0.704	0.711	0.744	0.706	0.724
Transcription rates [mol× L^{-1} × hours^{-1}]							
$V_{1_{\max}}$	*Per*	4.24e-11	4.03e-11	3.47e-11	3.45e-11	4.17e-11	3.44e-11

$V_{2_{\max}}$	*Cry*	3.79e-07	3.74e-07	3.79e-07	3.79e-07	3.77e-07	3.93e-07
$V_{3_{\max}}$	*Rev-Erb*	1.47e-09	1.49e-09	1.48e-09	1.47e-09	1.38e-09	1.53e-09
$V_{4_{\max}}$	*Ror*	1.64e-11	1.64e-11	1.62e-11	1.64e-11	1.64e-11	1.65e-11
$V_{5_{\max}}$	*Bmal*	3.27e-11	3.55e-11	3.81e-11	3.94e-12	4.84e-11	3.37e-11
$V_{6_{\max}}$	*Clock*	6.35e-11	6.01e-11	6.33e-11	5.11e-11	5.4e-11	6.33e-11
Activation/inhibition rates [mol$\times$ L^{-1}]							
k_{t_1}	*Per*-activation rate	4.68e-09	4.67e-09	4.55e-09	4.67e-09	2.35e-09	4.32e-09
k_{i_1}	*Per*-inhibition rate	1.25e-12	9.42e-13	1.25e-12	1.15e-12	5.29e-13	1.23e-12
k_{t_2}	*Cry*-activation rate	4.34e-09	4.35e-09	4.29e-09	4.33e-09	4.35e-09	4.37e-09
k_{i_2}	*Cry*-inhibition rate	1.92e-13	1.85e-13	1.02e-13	1.92e-13	1.59e-13	1.97e-13
$k_{i_{21}}$	*Cry*-inhibition rate	8.56e-07	8.54e-07	8.72e-07	8.58e-07	8.69e-07	8.55e-07
k_{t_3}	*Rev-Erb*-activation rate	2.06e-13	2.07e-13	2.04e-13	2.06e-13	2.06e-13	2.03e-13
k_{i_3}	*Rev-Erb*-inhibition rate	6.59e-12	7.79e-12	6.57e-12	3.26e-12	6.46e-12	6.94e-12

k_{t_4}	*Ror*-activation rate	9.62e-10	9.63e-10	9.58e-10	9.61e-10	1.08e-09	9.68e-10
k_{i_4}	*Ror*-inhibition rate	2.16e-13	2.16e-13	2.16e-13	2.16e-13	2.61e-13	2.14e-13
k_{t_5}	*Bmal*-activation rate	9.24e-06	7.76e-06	9.3e-06	9.24e-06	8.49e-06	9.23e-06
k_{i_5}	*Bmal*-inhibition rate	3.96e-07	3.17e-07	2.34e-07	2.17e-07	2.98e-07	7.72e-08
k_{t_6}	*Clock*-activation rate	1.03e-10	1.05e-10	1.06e-10	1.03e-10	1.04e-10	1.03e-10
k_{i_6}	*Clock*-inhibition rate	4.42e-08	3.95e-08	3.7e-08	2.17e-08	2.94e-08	9.26e-09
Transcription fold activation (dimensionless)							
a	*Per*	1.85e+02	1.89e+02	1.85e+02	1.84e+02	1.7e+02	1.78e+02
d	*Cry*	30.1	30.1	30.1	30.2	30.4	30.0
g	*Rev-Erb*	1.85e+02	1.91e+02	1.83e+02	1.85e+02	1.58e+02	1.85e+02
h	*Ror*	49.8	49.9	49.7	49.8	49.7	50.2
i	*Bmal*	3.99	3.99	3.68	3.99	3.98	3.97
j	*Clock*	68.7	68.9	54.0	68.5	68.6	68.5

Production rates [molecules × mRNA^{-1} × hour^{-1}]							
$\boldsymbol{k_{p_1}}$	PER_C^{tot}	1.14e+04	1.14e+04	1.14e+04	1.14e+04	1.09e+04	1.14e+04
$\boldsymbol{k_{p_2}}$	CRY_C	3.39e+04	3.41e+04	4.8e+04	3.15e+04	3.38e+04	3.29e+04
$\boldsymbol{k_{p_3}}$	REV-ERB_C	9.84e+04	8.18e+04	5.78e+04	1.57e+05	1.13e+05	8.7e+04
$\boldsymbol{k_{p_4}}$	ROR_C	1.2	1.2	1.19	1.2	1.2	1.27
$\boldsymbol{k_{p_5}}$	$BMAL_C$	7.45e+04	9.66e+04	7.21e+04	7.47e+04	7.66e+04	7.55e+04
$\boldsymbol{k_{p_6}}$	$CLOCK_C$	3.58e+02	3.58e+02	3.58e+02	3.58e+02	3.57e+02	3.66e+02
Import/Export rates [hour^{-1}]							
$\boldsymbol{k_{i_{z_4}}}$	PER/CRY_C^{tot}	0.00159	0.00159	0.00173	0.00158	0.00159	0.00161
$\boldsymbol{k_{i_{z_6}}}$	REV-ERB_C	2.67	2.67	2.69	2.67	2.68	2.67
$\boldsymbol{k_{i_{z_7}}}$	ROR_C	0.00118	0.00118	0.00133	0.00118	0.00119	0.00118
$\boldsymbol{k_{i_{z_9}}}$	CLOCK/$BMAL_C$	0.00094	0.000942	0.000947	0.000942	0.000938	0.00105
$\boldsymbol{k_{e_{x_1}}}$	CLOCK/$BMAL_N$	0.0161	0.0161	0.0122	0.0161	0.0161	0.0161
$\boldsymbol{k_{e_{x_2}}}$	PER/CRY_N^{tot}	0.34	0.337	0.493	0.34	0.234	0.385

Hill coefficients of transcription (dimensionless)							
$\boldsymbol{b}$	activation	1.35	1.36	1.19	1.22	1.45	1.73
$\boldsymbol{c}$	inhibition	1.45	1.47	1.44	1.46	1.44	1.63
$\boldsymbol{e}$	*Cry*-activation	7.62	7.5	7.9	6.76	7.11	7.85
$\boldsymbol{f}$	*Cry*-inhibition	7.71	7.98	7.98	7.8	7.71	7.97
$\boldsymbol{f_1}$	*Cry*-inhibition	2.37	2.31	2.26	2.38	2.39	2.4
Volume proportion (dimensionless)							
$\boldsymbol{v_c}$	cytoplasm	0.8	0.8	0.8	0.8	0.8	0.8
$\boldsymbol{v_n}$	nucleus	0.2	0.2	0.2	0.2	0.2	0.2
Transcription fold activation (dimensionless)							
$\boldsymbol{f_{\mathrm{Ppar}}}$	*PPAR*	32.0	32.0	32.1	32.1	32.2	31.7
$\boldsymbol{f_{\mathrm{Par}}}$	*PAR*	61.2	61.9	64.5	61.2	61.1	62.6
$\boldsymbol{f_{\mathrm{Ugt}}}$	*UGT*	34.7	34.8	34.6	34.6	35.1	55.3
$\boldsymbol{f_{\mathrm{Ces}}}$	*CES*	78.5	78.7	79.8	78.6	78.6	78.1
$\boldsymbol{f_{\mathrm{Nfil}}}$	*NFIL*	11.5	11.5	11.7	11.5	11.5	11.5
$\boldsymbol{f_{\mathrm{Abcb}}}$	*ABCB*	2.1e+02	2.11e+02	2.1e+02	2.16e+02	2.12e+02	2.09e+02

$f_{\mathbf{Abcc}}$	*ABCC*	1.17e+02	1.16e+02	1.16e+02	1.16e+02	68.5	92.0
Activation/inhibition rates [nmol× L^{-1}]							
$a_{\mathbf{Ppar}}$	*PPAR*	2.86e-08	2.63e-08	2.92e-08	2.86e-08	2.85e-08	4.98e-09
$i_{\mathbf{Ppar}}$	*PPAR*	1.9e-13	1.95e-13	1.94e-13	1.9e-13	1.9e-13	1.83e-13
$a_{\mathbf{Par}}$	*PAR*	1.44e-13	1.45e-13	1.43e-13	1.25e-13	1.45e-13	1.46e-13
$i_{\mathbf{Par}}$	*PAR*	1.84e-11	1.9e-11	2.28e-11	2.00e-11	1.81e-11	1.98e-11
$a_{\mathbf{Ugt}}$	*UGT*	1.75e-10	1.75e-10	1.75e-10	1.75e-10	6.06e-11	8.37e-11
$a_{\mathbf{Ces}}$	*CES*	1.11e-12	7.81e-13	1.11e-12	1.11e-12	1.03e-12	9.79e-13
$i_{\mathbf{Ces}}$	*CES*	1.6e-11	1.6e-11	1.65e-11	1.6e-11	1.68e-11	1.64e-11
$a_{\mathbf{Nfil}}$	*NFIL*	3.86e-11	3.85e-11	3.88e-11	3.86e-11	3.33e-11	3.92e-11
$i_{\mathbf{Nfil}}$	*NFIL*	3.07e-07	3.15e-07	3.37e-07	1.99e-07	3.08e-07	3.11e-07
$a_{\mathbf{Abcb}}$	*ABCB*	3.16e-09	3.17e-09	3.18e-09	3.16e-09	3.15e-09	3.16e-09
$i_{\mathbf{Abcb}}$	*ABCB*	1.46e-09	1.25e-09	1.46e-09	1.69e-09	1.46e-09	1.46e-09
$a_{\mathbf{Abcc}}$	*ABCC*	2.96e-09	2.96e-09	2.7e-09	2.96e-09	2.96e-09	3.07e-09
$i_{\mathbf{Abcc}}$	*ABCC*	5.57e-10	5.53e-10	5.53e-10	5.56e-10	3.59e-10	5.87e-10
$i_{\mathrm{Per}\mathbf{Nfil}}$	*PER inh. by* NFIL	0.00175	0.00175	0.00178	0.00175	0.00175	0.00175
i_{CesPpar}	*CES inh. by* PPAR	2.87e-11	2.86e-11	2.96e-11	2.86e-11	2.86e-11	2.89e-11

$\boldsymbol{i}_{\mathrm{Rev}\mathbf{Nfil}}$	*REV inh. by* NFIL	9.92e-06	9.94e-06	9.92e-06	1.00e-05	9.96e-06	9.92e-06
Transcription rates [nmol$\times$ $\boldsymbol{L}^{-1}$ $\times$ hours^{-1}]							
$\boldsymbol{V}_{\mathbf{Ppar}}$	*PPAR*	4.77e-12	4.78e-12	4.84e-12	5.63e-12	2.02e-12	4.79e-12
$\boldsymbol{V}_{\mathbf{Par}}$	*PAR*	5.5e-11	7.2e-11	5.99e-11	5.53e-11	5.47e-11	4.51e-11
$\boldsymbol{V}_{\mathbf{Ugt}}$	*UGT*	1.05e-13	1.05e-13	1.06e-13	1.05e-13	1.08e-13	5.67e-15
$\boldsymbol{V}_{\mathbf{Ces}}$	*CES*	3.25e-10	3.25e-10	3.29e-10	3.25e-10	3.28e-10	3.61e-10
$\boldsymbol{V}_{\mathbf{Nfil}}$	*NFIL*	7.16e-11	7.34e-11	4.64e-11	7.12e-11	6.16e-11	7.08e-11
$\boldsymbol{V}_{\mathbf{Abcb}}$	*ABCB*	1.22e-13	1.2e-13	1.22e-13	1.31e-13	1.22e-13	1.21e-13
$\boldsymbol{V}_{\mathbf{Abcc}}$	*ABCC*	3.22e-12	3.21e-12	3.16e-12	3.22e-12	1.78e-12	3.28e-12
Production rates [molecules $\times$ mRNA^{-1} $\times$ hour^{-1}]							
$\boldsymbol{r}_{\mathbf{PPAR}}$	PPAR	8.86	8.5	8.85	8.87	8.83	12.4
$\boldsymbol{r}_{\mathbf{PAR}}$	PAR	4.01e+02	2.71e+02	3.51e+02	4.01e+02	3.99e+02	4.00e+02
$\boldsymbol{r}_{\mathbf{NFIL}}$	NFIL	9.49e+02	1.04e+03	9.51e+02	9.47e+02	9.16e+02	8.49e+02
$r_{\mathrm{REV}*}$	REV activated	0.158	0.154	0.152	0.158	0.156	0.148
$r_{\mathrm{PAR}*}$	PAR activated	0.335	0.339	0.338	0.336	0.371	0.333
Degradation rates [hour^{-1}]							
$\boldsymbol{d}_{\mathbf{Ppar}}$	*PPAR*	0.131	0.131	0.0968	0.131	0.131	0.174

$\boldsymbol{d}_{\mathbf{PPAR}}$	PPAR	2.62	2.65	2.58	2.62	2.4	2.16
$\boldsymbol{d}_{\mathbf{Par}}$	*PAR*	0.4	0.4	0.421	0.349	0.431	0.526
$\boldsymbol{d}_{\mathbf{PAR}}$	PAR	0.115	0.124	0.115	0.115	0.0969	0.115
$\boldsymbol{d}_{\mathbf{Ugt}}$	*UGT*	1.69	1.77	1.69	1.74	1.1	1.69
$\boldsymbol{d}_{\mathbf{Ces}}$	*CES*	0.0373	0.0348	0.0373	0.0655	0.0373	0.0398
$\boldsymbol{d}_{\mathbf{Nfil}}$	*NFIL*	2.06	1.97	2.09	2.44	2.44	2.09
$\boldsymbol{d}_{\mathbf{NFIL}}$	NFIL	0.522	0.508	0.629	0.542	0.462	0.572
$\boldsymbol{d}_{\mathbf{Abcb}}$	*ABCB*	0.275	0.275	0.217	0.26	0.191	0.121
$\boldsymbol{d}_{\mathbf{Abcc}}$	*ABCC*	0.0325	0.0278	0.0344	0.0326	0.0323	0.0325
$\boldsymbol{d}_{\mathrm{REV*}}$	REV activated	0.395	0.389	0.403	0.395	0.398	0.416
$\boldsymbol{d}_{\mathrm{PAR*}}$	PAR activated	0.783	0.619	0.524	0.725	0.732	0.85
Circadian degradation parameters (dimensionless)							
$A_{\mathrm{REV*}}$	REV activated	0.0236	0.0236	0.0236	0.0238	0.0235	0.0234
$\phi_{\mathrm{REV*}}$	REV activated	1.2e+02	1.17e+02	1.15e+02	1.26e+02	1.17e+02	1.07e+02
$A_{\mathrm{PAR*}}$	PAR activated	0.302	0.298	0.3	0.301	0.301	0.303
$\phi_{\mathrm{PAR*}}$	PAR activated	1.26e+02	85.4	1.11e+02	1.26e+02	1.2e+02	1.25e+02
